# Metabolic syndrome and its components among obese (BMI ≥95th) Mexican adolescents

**DOI:** 10.1530/EC-13-0057

**Published:** 2013-11-18

**Authors:** Maria Lola Evia-Viscarra, Edel Rafael Rodea-Montero, Evelia Apolinar-Jiménez, Silvia Quintana-Vargas

**Affiliations:** 1 Pediatric Endocrinology DepartmentHospital Regional de Alta Especialidad del BajíoBoulevard Milenio 130, San Carlos la RonchaC.P. 37670, León, GuanajuatoMexico; 2 Research DepartmentHospital Regional de Alta Especialidad del BajíoBoulevard Milenio 130, San Carlos la RonchaC.P. 37660, León, GuanajuatoMexico

**Keywords:** adolescents, dyslipidemia, hypertension, metabolic syndrome, obesity

## Abstract

The aim of this study was to estimate the prevalence of metabolic syndrome (MS) and its components in obese Mexican adolescents and to compare the clinical, anthropometric, and biochemical characteristics between patients with and without MS by sex. We conducted a cross-sectional study with a sample of 110 obese adolescents (boys and girls) from 8 to 16 years old (BMI ≥95th percentile), who were recruited in the pediatric obesity clinic of a third-level care hospital. A frequency analysis was used to estimate the prevalence of MS and its components, and the assessments were compared between the sexes and between the groups with and without MS using the Kruskal–Wallis test. The prevalence of MS was 62%. In order of prevalence, the following components of MS were observed in the sample: abdominal obesity (88%), high triglycerides (TG) (85%), low HDL-C (60%), hypertension (35%), and hyperglycemia (5%). In the groups with MS, hypertension (*P*<0.001), waist circumference (*P*=0.003), and TG (*P*=0.012) were significantly higher, and HDL-C (*P*<0.001) was significantly lower. In conclusion the prevalence of MS and its components is high among obese Mexican-Hispanic children. These findings show the importance of preventing and treating obesity in the early stages of life in order to decrease the incidence rates of cardiovascular disease and type 2 diabetes mellitus.

## Introduction

A sedentary lifestyle and a diet rich in carbohydrates and fats during childhood leads to obesity. In Mexico, the National Health and Nutrition Survey 2012 data estimate a high prevalence of obesity, defined as a BMI ≥ the 95th percentile, at 14.6% among children (5–11 years) and 13.3% among adolescents (12–19 years) (1). These prevalence rates are similar to those reported in other high-prevalence countries (2). Pediatric obesity is accompanied by various conditions: orthopedic, respiratory, metabolic (hyperglycemia, dyslipidemia), hepatic (non-alcoholic fatty liver disease), and cardiovascular disease (CVD) (arterial hypertension and vascular dysfunction). The chronic evolution of obesity generates devastating consequences that are associated with long periods of physical incapacity and early mortality (3).

The risk factors for developing CVD and type 2 diabetes mellitus (T2DM) in adults are encompassed by the term ‘metabolic syndrome’ (MS) (4), which is diagnosed based on the criteria of the National Cholesterol Education Program (NCEP) Adult Treatment Panel III (ATP III) (4). Subsequently, these criteria were modified for the pediatric population by obtaining representative values for children. The initial research on the general pediatric population determined that MS may be present in 4.2 to 12.9% of children. The reported prevalence of MS in obese adolescents ranged from 28.7 to 38.1%, depending on the ethnic group considered or the use of percentiles or absolute value cutoffs to determine the presence of each component of MS (5, 6, 7, 8).

MS is not only a clinical disease precursor but is also currently associated with vascular dysfunction and T2DM in children (9). There is evidence that these risk factors accelerate the development of early atherosclerosis beginning in childhood (10). In both children and adults with obesity and MS, insulin resistance (IR) and chronic inflammation appear to be responsible for the changes that induce complications or comorbidities (11).

In addition, Mexican-Hispanic children are recognized as a high-risk ethnic group (1), which indicates that there will be an increase in the dramatic consequences of obesity (CVD and T2DM) in this population in a few years. Therefore, an investigation of the presence of MS in obese patients in the early stages of life is necessary to plan public health programs for prevention and treatment.

The aim of this study was to estimate the prevalence of MS and its components in obese adolescents and to compare the clinical, anthropometric, and biochemical characteristics between patients with and without MS by sex.

## Subjects and methods

### Participants

We conducted a cross-sectional study with a sample of obese adolescents (boys and girls) from 8 to 16 years old, who were recruited in the Pediatric Obesity Clinic of the third-level Mexican High-Specialty Regional Bajío Hospital (Hospital Regional de Alta Especialidad del Bajío, HRAEB), located in León City in Guanajuato state (México), between April 2008 and December 2012. All of the participants were Mexican-Hispanic and had BMI values ≥ the 95th percentile for their age and sex according to the tables published by the Centers for Disease Control and Prevention (CDC). The exclusion criteria were secondary obesity syndromes, obesity associated with the use of medication, and concomitant chronic illnesses.

### Approvals

The protocol for this study was reviewed and accepted by the Research and Ethics Committees of the HRAEB. The patients' parents signed a written consent form when they and their adolescent agreed to enroll.

### Clinical assessments

A complete physical examination was conducted, and blood pressure and pubertal stage according to the Tanner classification were evaluated by a pediatric endocrinologist. Systolic and diastolic blood pressure (SBP and DBP respectively) were taken twice after the participant had been sitting comfortably for 5 min, using a mercury sphygmomanometer (Riester, Jungingen, Germany) with an appropriately sized cuff on the right arm, which was slightly flexed at heart level. The two-measurement average was used for the analysis. Hypertension (elevated SBP or DBP) was defined as a value that exceeded the 90th percentile for sex, age, and height or the use of anti-hypertensive medication (12).

### Anthropometric assessments

The anthropometric assessments included weight (kg), height (cm), and waist circumference (WC, cm). All of these indicators were evaluated by a pediatric nutritionist. Weight was measured with the children wearing light clothes and no shoes while standing upright at the center of the platform scale. Height was measured using a stadiometer (Seca, Hamburg, Germany) with the children standing in an upright position without shoes, with heels together, their arms extended down on both sides of the body, and their head positioned parallel to the floor. WC was measured using a measuring tape (Seca), at the level of the midpoint between the lower costal border and the iliac crest at the end of a normal expiration (13). Height and WC were measured to the nearest 0.1 cm, and weight was measured to the nearest 0.1 kg. The BMI was calculated by dividing the weight in kilograms by the square of the height in meters. The weight, height, and BMI percentiles were assessed according to the CDC criteria. The WC percentiles were assessed using Mexican-Hispanic tables for children and adolescents (14).

### Biochemical assessments

The serum samples were processed in an HRAEB Laboratory by the same individual, who had no knowledge about the clinical details of the patients. An oral glucose tolerance test (OGTT) was performed following an 8–10 h fasting period (1.75 g/kg, maximum 75 g anhydrous glucose). The glucose measurements included fasting plasma glucose (FPG) and a 2-h value after the dose of oral glucose. Serum glucose was measured using colorimetric glucose oxidase slides (Vitros 350; Ortho Clinical Diagnostics, Johnson & Johnson, Raritan, NJ, USA). The interassay coefficient of variation was 3.49, and the s.d. was 3.04%.

Insulin was determined using an ELISA (Monobind Inc., Lake Forest, CA, USA). The homeostasis model assessment (HOMA) index was calculated as the concentration of fasting insulin (μU/ml) multiplied by the concentration of FPG (mmol/l)/22.5, assuming that adolescents have IR when HOMA ≥3.16 (15).

Serum total cholesterol, HDL-C, LDL-C, and triglycerides (TG) were measured using dry chemistry with colorimetric methods (Vitros 350; Ortho Clinical Diagnostics, Johnson & Johnson).

### Definition of MS

MS in the pediatric population was diagnosed according to the modified ATP III criteria of Cruz *et al*. (6) using the percentile values for age and sex (Table 1). MS is defined as having at least three of the following five abnormalities: abdominal obesity measured via WC, elevated TG, low HDL-C, hypertension, or if subjects reported current use of any antihypertensive drugs, and hyperglycemia (4). According to the recent recommendation of the American Diabetes Association (ADA), we defined hyperglycemia as an FPG level ≥5.6 mmol/l (≥100 mg/dl), impaired glucose tolerance as a glucose level ≥7.8 mmol/l (≥140 mg/dl) but <11.1 mmol/l (200 mg/dl) 2-h after the OGTT, and T2DM as an FPG ≥7.0 mmol/l (≥126 mg/dl) or ≥11.1 mmol/l (200 mg/dl) 2-h after the OGTT (16).

### Statistical analysis

All of the data were analyzed using the statistical software R (17). A frequency analysis was used to estimate the prevalence of MS and its components, and the association between MS and sex was also tested using the *χ*^2^ test. Descriptive statistics were calculated for the patients' anthropometric and biochemical results. These results were compared between the sexes and between the groups with and without MS using the Kruskal–Wallis test. The sample size allows for the detection of a difference of ≥10% in any assessment (with type I error *α*=0.05 and type II error *β*=0.80). In all cases, 95% CIs were constructed, and a statistical significance level of *α*=0.05 was used.

## Results

**Figure 1:**
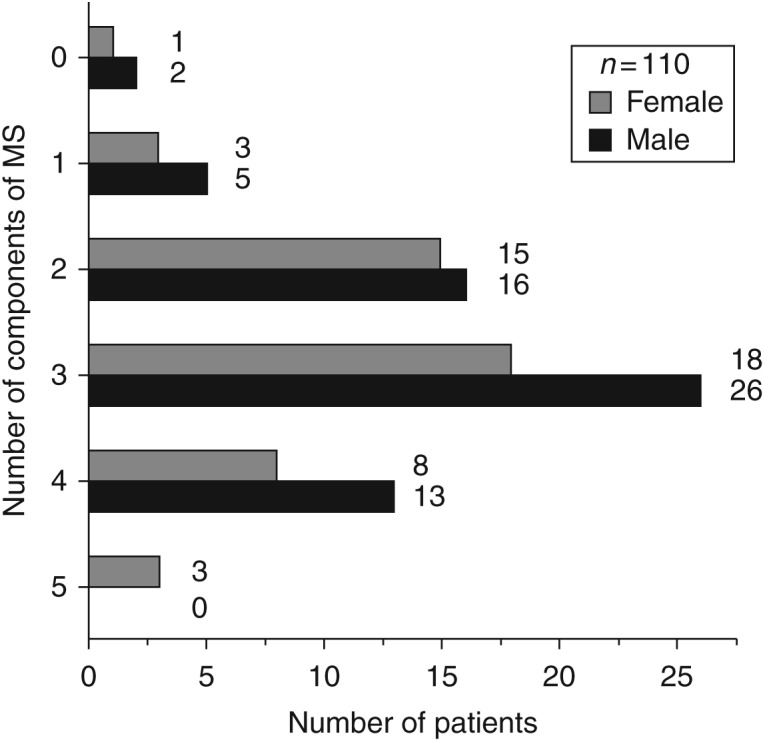
The study patients grouped according to sex and the number of metabolic syndrome components.

The final analysis included 110 obese adolescents: 48 (44%) females and 62 (56%) males. The mean (±s.d.) age of the patients was 11.55±2.02 years (range 8.11–15.97 years). The presence of MS and its components in the study population is shown in Table 2. Overall, the prevalence of MS among the patients was 62%. In order of prevalence, the following components of MS were observed in the sample: abdominal obesity (88%), high TG (85%), low HDL-C (60%), hypertension (35%), and hyperglycemia (5%). There were no significant differences between males and females for any of the components. In addition, we analyzed a number of the components of MS by sex in detail (Fig. 1) and found that three patients (one female and two males) did not demonstrate any components of MS despite having a BMI ≥ the 95th percentile. Another important result was that only three female patients had all the five components of MS.

The patients were divided into four groups by sex and the presence (+) or absence (−) of MS. The clinical and anthropometric measurements are shown in Table 3. Based on the inter-group comparison, the ages of the groups appeared to be similar (*P*=0.802), indicating that the groups were homogenous with regard to age. The median Tanner classification was significantly different (*P*<0.001) between the females (median Tanner score of 3) and males (median Tanner score of 2). The SBP and DBP values were significantly higher (*P*<0.001 in both cases) in the groups with MS. There were apparent but not significant differences in the BMI (*P*=0.054) and BMI percentile (*P*=0.054) between the patients with and without MS. Weight was significantly higher in the groups with MS (*P*=0.023). Height was significantly higher in the groups with MS (*P*=0.043). There were also significant differences in the WC (*P*=0.003): the patients with MS had higher values.

Table 4 illustrates the biochemical characteristics of the study population. There was no evidence of differences among the groups in the glucose metabolism assessments. The mean FPG and 2-h glucose values appeared to be similar and unaltered across all groups (*P*=0.325 and *P*=0.180, respectively). The adolescents with MS, especially the females, had slightly higher serum insulin concentrations, although no significant differences were detected (*P*=0.345). With regard to HOMA values, there were no significant differences among the four groups; however, the MS groups presented values above normal, indicating that these patients had some degree of IR, especially the adolescent females with MS. With regard to the lipid profile, there were no significant differences among the groups in their cholesterol and LDL-C levels. The comparisons of TG showed significantly higher (*P*=0.012) values in the patients with MS, particularly the females. HDL-C was significantly lower in the MS groups (*P*<0.001).

## Discussion

Most studies published in the literature on MS in the pediatric population compare normal-weight, overweight (85th ≤ BMI <95th), and obese (BMI ≥95th) children. This study analyzed the presence of the components of MS specifically in obese adolescents and excluded normal-weight or overweight children. Therefore, this work, unlike most studies on pediatric MS, enables a comparison of obese adolescents with and without MS who share similar lifestyles and dietary habits.

The ages of the participants were not significantly different among the groups. Overall, the children were in puberty, but the girls showed more advanced Tanner stages, most probably because females start puberty at an earlier age than males. This difference could also be related to the fact that excess adiposity in girls may influence the early onset of puberty, as some authors have suggested (18).

Previous studies appeared to show a greater prevalence of MS among males. Our study did not find significant differences in the presence of MS or its components between males and females, which could be related to the different Tanner stages.

The estimated prevalence of MS in this study was 62%, higher than the global reports that have estimated the prevalence between at 28.7 and 30.7% among children with obesity (5, 19) and between at 30 and 35.8% in groups in which overweight and obese children are combined (5, 6, 7, 20). Our results are similar to the results of studies conducted in Mexican populations; for example, the study by Elizondo-Montemayor *et al*. (21) reported that the prevalence of MS based on BMI *Z*-scores ranged from 42.6 (2<*Z*≤2.5) to 73.6% (*Z*>2.5). In comparison, we found a prevalence of 79.2% among the patients whose BMI *Z*-score was >2.5. In accordance with a study conducted in the United States of America among Mexican-American children, our population had a high prevalence of MS and therefore high cardiovascular metabolic risk (5).

In our study, three of the children did not show any components of MS, even though their BMI values indicated the presence of obesity. This result makes us question whether BMI is a predictor of MS for all obese pediatric patients. We used the WC reference tables developed specifically for the Mexican population by Klunder-Klunder & Flores-Huerta (14), and when we compared the 90th percentile of WC with the proposals in the study of Fernández *et al*. (22), we found that the concordance was >95%. Central obesity was significantly more common in the MS groups, supporting the observation that WC is a better predictor of MS than BMI. Lee *et al*. showed that there is a strong correlation among WC, dyslipidemia, and hypertension. This correlation is associated with increased abdominal visceral fat metabolism, which appears to promote lipid disorders and IR, two factors that are responsible for the deleterious effects of obesity (23).

The prevalence of hypertriglyceridemia in obese children with MS was very high compared with worldwide studies on adolescents (6, 7) and even compared with other studies on Hispanic populations (85 vs 20–30%) (20). The prevalence of low HDL-C in the groups with MS is similar to the prevalence reported in other studies (50 vs 53.7%) (6, 20).

Hypertriglyceridemia combined with low HDL-C levels has been strongly linked with the presence of IR (24) and is associated with increased central adipose tissue (25). In this study, we observed that the patients with MS had greater WC than the obese adolescents without MS. Some authors have proposed that there is a strong association between TG/HDL-C and IR in adolescents and that this indicator could be used to assess the CVD risk in obese children (26). Autopsy studies and vascular function studies (27) in obese children have strongly indicated that abnormal lipid levels are associated with an increased incidence of atherosclerosis.

Dyslipidemia among the children with MS in this study can be attributed not only to a genetic predisposition but also to the typical diet in this country.

The prevalence of hypertension in our study population was higher than that in other studies (35 vs 20.6%) (20). Some studies have identified hypertension in obese children as the factor that is responsible for the genesis of atherosclerosis, vascular dysfunction, CVD, and premature death (3, 28, 29).

The insulin levels were slightly but not significantly higher in the groups with MS, but the mean glucose level (before and after OGTT) was normal. The mean insulin level was >104.18 pmol/l (15 μIU/ml), with wide variability; this value was proposed as an indicator of IR by Reaven (30). The HOMA index was also higher in the MS groups, especially in the girls (HOMA>4). Currently, there is no consensus on the appropriate HOMA cutoff for defining IR in adolescents. Keskin *et al*. (31) defined IR as a HOMA value ≥3.6, but recently, some authors (32, 33) have preferred to use the cutoff of 3.16 for predicting MS, as suggested by Tresaco *et al*. (15). The insulin sensitivity is abnormal for all of the subjects in our study population, considering that the HOMA level in normal young people is close to 1 (34).

The frequency of hyperglycemia in this study was lower than the frequency observed by Shaibi & Goran (5 vs 25.2%) in a Hispanic population (20) and even lower than those reported among Mexican groups (35). This low prevalence may be attributed to the fact that our patients had previously been evaluated in a primary care office; diabetes mellitus is usually scrutinized carefully in all obese patients given the alarming growth of T2DM in Mexican children (36). However, this prevalence of hyperglycemia, although relatively low compared with the other components of MS, indicates that the compensatory mechanism caused by hyperinsulinism is no longer sufficient and that pancreatic function deteriorates in the early stages of life. Thus, prospective studies of MS in children should be performed to elucidate the risk factors and the time needed for their condition to lead to the deterioration of pancreatic β-cell function. Some authors consider MS to be a poor predictor of T2DM (37) and even suggest that haemaglobin A1c may be a more sensitive indicator to predict the loss of pancreatic function (38). Based on this reasoning, the MS components should not be evaluated in a dichotomous way; instead, each component should be studied to determine its possible value for predicting CVD or T2DM.

Moreover, there are no unified worldwide criteria for determining the presence of MS in children. Although the International Diabetes Federation Workshop (39) tried to unify the criteria, it only considered children older than 10 years and created absolute values for each of the parameters. We found it difficult to follow these recommendations because this approach would limit research on obese children under 10 years old, a group that could experiences complications (9, 40). Using the ATP III criteria modified by Cruz *et al*. (6) allowed us to stratify each component and better evaluate our population.

This study has certain limitations. It was a cross-sectional study, and thus, causality cannot be inferred. The subjects in this study were not recruited from the general population; they were referred to our center because they were identified as a high-risk population in primary and secondary health care offices. This sample consisted of Mexican children, which may limit its generalizability to other ethnic groups. Some critics might suggest that the study lacks a control group of healthy children. However, the goal of our study was to assess the presence of MS and its components in obese children with increased metabolic risk to pay specific attention to them and to design more effective treatment measures.

## Conclusion

Our study showed that the prevalence of MS is high among obese Mexican-Hispanic children. A large WC, which influences the changes in TG, HDL-C, and arterial hypertension, is particularly common. In the coming years, this alteration will lead to increases in the incidence rates of CVD and T2DM in our country. Therefore, the high prevalence of MS in obese children shows the importance of preventing and treating obesity in a timely manner beginning in the early stages of life. Our results also indicate that hypertriglyceridemia is highly prevalent among children with obesity and MS in this ethnic group. Further studies are required to assess the etiology, evolution, and consequences of this alteration.

## Figures and Tables

**Table 1:** Criteria for metabolic syndrome.

**Criterion**	**Adults, ATP III**	**Children, ATP III modified by Cruz *et al*.**
High TG level	≥1.7 mmol/l (≥150 mg/dl)	≥90th percentile (age- and sex-specific)
Low HDL-C level	Men ≤1.1 mmol/l (≤40 mg/dl)	≤10th percentile (age- and sex-specific)
	Women ≤1.3 mmol/l (≤50 mg/dl)	
Abdominal obesity (via WC)	Men >102 cm	≥90th percentile (age-, sex-, and race-specific)
	Women >88 cm	
High FPG level	≥5.6 mmol/l (≥100 mg/dl)	≥5.6 mmol/l (≥100 mg/dl)
Hypertension	SBP ≥130 mmHg, DBP ≥85 mmHg or using treatment for previously diagnosed hypertension	≥90th percentile (age-, sex-, and height-specific)

**Table 2:** The presence of metabolic syndrome and its components in the study population by sex.

	**Overall** (*n*=110)	**Female** (*n*=48)	**Male** (*n*=62)	**Inter-group comparison**^a^
Metabolic syndrome, *n* (%)	68 (62%)	29 (60%)	39 (63%)	*P*=0.790
High TG, *n* (%)	94 (85%)	43 (90%)	51 (82%)	*P*=0.260
Low HDL-C, *n* (%)	66 (60%)	28 (58%)	38 (61%)	*P*=0.754
Abdominal obesity, *n* (%)	97 (88%)	42 (88%)	55 (89%)	*P*=0.845
Hyperglycemia, *n* (%)	5 (5%)	3 (6%)	2 (3%)	*P*=0.450
Hypertension, *n* (%)	39 (35%)	18 (38%)	21 (34%)	*P*=0.693

a *χ*^2^ test with 1 degree of freedom.

**Table 3:** Clinical and anthropometric characteristics of the study population grouped by sex and the presence of metabolic syndrome.

	**Overall** (*n*=110)	**Female** (*n*=48)		**Male** (*n*=62)		**Inter-group comparison**^a^
Without MS (−) (*n*=19)	With MS (+) (*n*=29)	Without MS (−) (*n*=23)	With MS (+) (*n*=39)
Clinical						
Female, *n* (%)	48 (44%)					
Male, *n* (%)	62 (56%)					
Age (years)	11.55 (2.02)	11.54 (2.6)	11.84 (1.92)	11.3 (1.85)	11.48 (1.91)	*P*=0.802
Tanner (median)	2	3	3	2	2	*P*<0.001^b^
SBP (mmHg)	111.11 (10.43)	107.11 (6.52)	113.41 (7.77)	104.74 (10.47)	115.1 (11.44)	*P*<0.001^b^
DBP (mmHg)	65.51 (9.54)	63.16 (8.85)	70.48 (8.67)	59.48 (8.52)	66.51 (9.05)	*P*<0.001^b^
Anthropometric						
Weight (kg)	70.26 (19.25)	65.19 (17.29)	75.28 (20.19)	61.89 (16.52)	73.93 (19.35)	*P*=0.023^b^
Height (cm)	149.89 (11.6)	147.04 (10.75)	152.34 (10.55)	145.63 (10.9)	151.96 (12.51)	*P*=0.043^b^
BMI (kg/m^2^)	30.6 (5.3)	29.62 (5.49)	31.77 (5.22)	28.38 (4.67)	31.51 (5.33)	*P*=0.054
BMI percentile	98.49 (1.04)	98.27 (0.95)	98.54 (1.18)	98.18 (1.05)	98.76 (0.93)	*P*=0.054
WC (cm)	93.76 (11.43)	87.35 (8.84)	95.49 (11.15)	90.15 (11.1)	97.73 (11.31)	*P*=0.003^b^

Unless otherwise indicated, the values are given as the mean (s.d.).

a Kruskal–Wallis test with three degrees of freedom.

b Significant *P* values.

**Table 4:** Biochemical characteristics of the study population grouped by sex and the presence of metabolic syndrome.

	**Overall** (*n*=110)		**Female** (*n*=48)				**Male** (*n*=62)				**Inter-group comparison**^a^
Without MS (−) (*n*=19)		With MS (+) (*n*=29)		Without MS (−) (*n*=23)		With MS (+) (*n*=39)	
Metabolic											
FPG (mmol/l)	4.83 (0.38)		4.71 (0.27)		4.85 (0.48)		4.87 (0.32)		4.87 (0.36)		*P*=0.325
Glucose 2-h (mmol/l)	5.81 (1.23)	*n*=103	6.12 (1.23)	*n*=18	5.87 (1.33)	*n*=27	5.32 (1.02)	*n*=21	5.91 (1.24)	*n*=37	*P*=0.180
Fasting insulin (pmol/l)	115.91 (122.72)		84.03 (66.46)		152.44 (145.57)		101.54 (82.92)		112.65 (140.84)		*P*=0.345
HOMA	3.62 (3.95)		2.54 (2.04)		4.83 (4.88)		3.15 (2.62)		3.51 (4.4)		*P*=0.308
Lipid profile											
Cholesterol (mmol/l)	4.31 (0.85)		4.22 (0.53)		4.49 (1.02)		4.53 (0.82)		4.07 (0.82)		*P*=0.145
TG (mmol/l)	1.98 (0.91)		1.69 (0.66)		2.37 (0.91)		1.69 (0.83)		2.00 (0.98)		*P*=0.012^b^
HDL-C (mmol/l)	0.98 (0.25)		1.14 (0.32)		0.89 (0.15)		1.15 (0.21)		0.87 (0.19)		*P*<0.001^b^
LDL-C (mmol/l)	2.43 (0.7)		2.34 (0.56)		2.56 (0.85)		2.62 (0.66)		2.28 (0.66)		*P*=0.356

Unless otherwise indicated, the values are given as the mean (s.d.).

a Kruskal–Wallis test with three degrees of freedom.

b Significant *P* values.

## References

[1] Gutierrez JP, Rivera Dommarco J, Shamah Levy T, Villalpando Hernández S, Franco A, Cuevas Nasu L, Romero Martínez M & Hernández Ávila M. 2012 *Resultados Nacionales*. Cuernavaca, México: Encuesta Nacional de Salud y Nutrición.

[2] de Onis M, Blössner M, Borghi E (2010). Global prevalence and trends of overweight and
obesity among preschool children. American Journal of
Clinical
Nutrition.

[3] Franks PW, Hanson RL, Knowler WC, Sievers ML, Bennett PH, Looker HC (2010). Childhood obesity, other cardiovascular risk
factors, and premature death. New England Journal of
Medicine.

[4] National Cholesterol Education Program
(NCEP) Expert Panel on Detection, Evaluation, and Treatment of High Blood
Cholesterol in Adults (Adult Treatment Panel
III) (2002). Third Report of the National Cholesterol
Education Program (NCEP) Expert Panel on Detection, Evaluation, and
Treatment of High Blood Cholesterol in Adults (Adult Treatment Panel III)
final
report. Circulation.

[5] Cook S, Weitzman M, Auinger P, Nguyen M, Dietz WH (2003). Prevalence of a metabolic syndrome phenotype in
adolescents: findings from the third National Health and Nutrition
Examination Survey, 1988–1994. Archives of
Pediatrics & Adolescent
Medicine.

[6] Cruz M, Weigenserg M, Ball G, Shaibi G, Goran M (2004). The metabolic syndrome in overweight Hispanic
yougth and the role of insulin sensitivity. Journal of
Clinical Endocrinology and
Metabolism.

[7] de Ferranti SD, Gauvreau K, Ludwig DS, Neufeld EJ, Newburger JW, Rifai N (2004). Prevalence of the metabolic syndrome in American
adolescents: findings from the Third National Health and Nutrition
Examination
Survey. Circulation.

[8] Weiss R, Sziura J, Burgert T, Tamborlane W, Taksali S (2004). Obesity and the metabolic syndrome in children and
adolescents. New England Journal of
Medicine.

[9] Liese AD, D'Agostino RB, Hamman RF, Kilgo PD, Lawrence JM, Liu LL, Loots B, Linder B, Marcovina S, Rodriguez B (2006). The burden of diabetes mellitus among US youth:
prevalence estimates from the SEARCH for Diabetes in Youth
Study. Pediatrics.

[10] Beauloye V, Zech F, Tran HP, Clapuyt P, Maes M (2007). Determinants of early atherosclerosis in obese
children and adolescents. Journal of Clinical
Endocrinology and
Metabolism.

[11] Herder C, Schneitler S, Rathmann W, Haastert B, Schneitler H, Winkler H, Bredahl R, Hahnloser E, Martin S (2007). Low-grade inflammation, obesity, and insulin
resistance in adolescents. Journal of Clinical
Endocrinology and
Metabolism.

[12] National High Blood Pressure Education
Program Working Group on High Blood Pressure in Children and
Adolescents (2004). The Fourth Report on the Diagnosis,
Evaluation, and Treatment of High Blood Pressure in Children and
Adolescents. Pediatrics.

[13] McCarthy HD, Jarrett KV, Emmett PM, Rogers I (2005). Trends in waist circumferences in young British
children: a comparative study. International Journal
of
Obesity.

[14] Klunder-Klunder M, Flores-Huerta S (2011). Waist circumference values according to height
percentiles: a proposal to evaluate abdominal obesity in Mexican children
and adolescents between 6 and 16 years of
age. Archives of Medical
Research.

[15] Tresaco B, Bueno G, Pineda I, Moreno LA, Garagorri JM, Bueno M (2005). Homeostatic model assessment (HOMA) index cut-off
values to identify the metabolic syndrome in
children. Journal of Physiology and
Biochemistry.

[16] American Diabetes
Association (2012). Diagnosis and classification of diabetes
mellitus. Diabetes
Care.

[17] R Development Core Team. 2006 *A language and environment for statistical computing*. Vienna, Austria: Foundation for Statistical Computing.

[18] Roemmich JN, Clark PA, Lusk M, Friel A, Weltman A, Epstein LH, Rogol AD (2002). Pubertal alterations in growth and body
composition. VI. Pubertal insulin resistance: relation to adiposity, body
fat distribution and hormone release. International
Journal of Obesity and Related Metabolic
Disorders.

[19] Andrabi SM, Bhat MH, Andrabi SR, Kamili MM, Imran A, Nisar I, Nisar U (2013). Prevalence of metabolic syndrome in
8–18-year-old school-going children of Srinagar city of Kashmir
India. Indian Journal of Endocrinology and
Metabolism.

[20] Shaibi GQ, Goran MI (2008). Examining metabolic syndrome definitions in
overweight Hispanic youth: a focus on insulin
resistance. Journal of
Pediatrics.

[21] Elizondo-Montemayor L, Serrano-Gonzalez M, Ugalde-Casas PA, Bustamante-Careaga H, Cuello-Garcia C (2011). Waist-to-height: cutoff matters in predicting
metabolic syndrome in Mexican children. Metabolic
Syndrome and Related
Disorders.

[22] Fernández JR, Redden DT, Pietrobelli A, Allison D (2004). Waist circumference percentiles in nationally
representative samples of African-American, European-American, and
Mexican-American children and adolescents. Journal of
Pediatrics.

[23] Lee S, Bacha F, Gungor N, Arslanian SA (2006). Waist circumference is an independent predictor of
insulin resistance in black and white youths. Journal
of
Pediatrics.

[24] Steinberger J, Moorehead C, Katch V, Rocchini AP (1995). Relationship between insulin resistance and
abnormal lipid profile in obese adolescents. Journal
of
Pediatrics.

[25] Freedman DS, Mei Z, Srinivasan SR, Berenson GS, Dietz WH (2007). Cardiovascular risk factors and excess adiposity
among overweight children and adolescents: the Bogalusa Heart
Study. Journal of
Pediatrics.

[26] Giannini C, Santoro N, Caprio S, Kim G, Lartaud D, Shaw M, Pierpont B, Weiss R (2011). The triglyceride-to-HDL cholesterol ratio:
association with insulin resistance in obese youths of different ethnic
backgrounds. Diabetes
Care.

[27] Koopman LP, McCrindle BW, Slorach C, Chahal N, Hui W, Sarkola T, Manlhiot C, Jaeggi ET, Bradley TJ, Mertens L (2012). Interaction between myocardial and vascular
changes in obese children: a pilot study. Journal of
the American Society of
Echocardiography.

[28] Juonala M, Viikari JS, Rönnemaa T, Helenius H, Taittonen L, Raitakari OT (2006). Elevated blood pressure in adolescent boys
predicts endothelial dysfunction: the Cardiovascular Risk in Young Finns
Study. Hypertension.

[29] Aggoun Y, Farpour-Lambert NJ, Marchand LM, Golay E, Maggio AB, Beghetti M (2008). Impaired endothelial and smooth muscle functions
and arterial stiffness appear before puberty in obese children and are
associated with elevated ambulatory blood
pressure. European Heart
Journal.

[30] Reaven GM (1988). Role of insulin resistance in human
disease. Diabetes.

[31] Keskin M, Kurtoglu S, Kendirci M, Atabek M, Yazici C (2005). Homeostasis model assessment is more reliable than
the fasting glucose/insulin ratio and quantitative insulin sensitivity check
index for assessing insulin resistance among obese children and
adolescents. Pediatrics.

[32] Khoury M, Manlhiot C, McCrindle B (2013). Role of the waist/height ratio in the
cardiometabolic risk assessment of children classified by body mass
index. Journal of the American College of
Cardiology.

[33] Agirbasli M, Agaoglu NB, Orak N, Caglioz H, Ocek T, Poci N, Salaj A, Maya S (2009). Sex hormones and metabolic syndrome in children
and adolescents. Metabolism: Clinical and
Experimental.

[34] Matthews DR, Hosker JP, Rudenski AS, Naylor BA, Treacher DF, Turner RC (1985). Homeostasis model assessment: insulin resistance
and β-cell function from fasting plasma glucose and insulin
concentrations in
man. Diabetologia.

[35] Guerrero-Romero F, Violante R, Rodríguez-Morán M (2009). Distribution of fasting plasma glucose and
prevalence of impaired fasting glucose, impaired glucose tolerance and type
2 diabetes in the Mexican paediatric
population. Paediatric and Perinatal
Epidemiology.

[36] Cruz M, Torres M, Aguilar-Herrera B, Perez-Johnston R, Guzman-Juarez N, Aranda M, Kumate J (2004). Type 2 diabetes mellitus in children – an
increasing health problem in Mexico. Journal of
Pediatric Endocrinology &
Metabolism.

[37] Hwang YC, Jee JH, Oh EY, Choi YH, Lee MS, Kim KW, Lee MK (2009). Metabolic syndrome as a predictor of
cardiovascular diseases and type 2 diabetes in
Koreans. International Journal of
Cardiology.

[38] Cheng P, Neugaard B, Foulis P, Conlin PR (2011). Hemoglobin A1c as a predictor of incident
diabetes. Diabetes
Care.

[39] Zimmet P, Alberti KG, Kaufman F, Tajima N, Silink M, Arslanian S, Wong G, Bennett P, Shaw J, Caprio S (2007). The metabolic syndrome in children and adolescents
– an IDF consensus report. Pediatric
Diabetes.

[40] Watts K, Beye P, Siafarikas A, O'Driscoll G, Jones TW, Davis EA, Green DJ (2004). Effects of exercise training on vascular function
in obese children. Journal of
Pediatrics.

